# Transcriptomic analyses reveal the adaptive features and biological differences of guts from two invasive whitefly species

**DOI:** 10.1186/1471-2164-15-370

**Published:** 2014-05-15

**Authors:** Xiao-Dong Ye, Yun-Lin Su, Qiong-Yi Zhao, Wen-Qiang Xia, Shu-Sheng Liu, Xiao-Wei Wang

**Affiliations:** Ministry of Agriculture Key Laboratory of Agricultural Entomology, Institute of Insect Sciences, Zhejiang University, 866 Yuhangtang Road, Hangzhou, 310058 China; The University of Queensland, Queensland Brain Institute, Brisbane, Qld 4072 Australia

**Keywords:** Gene expression, Genetic divergence, Gut, Transcriptome, Whitefly

## Abstract

**Background:**

The gut of phloem feeding insects is critical for nutrition uptake and xenobiotics degradation. However, partly due to its tiny size, genomic information for the gut of phloem feeding insects is limited.

**Results:**

In this study, the gut transcriptomes of two species of invasive whiteflies in the *Bemisia tabaci* complex, Middle East Asia Minor 1 (MEAM1) and Mediterranean (MED), were analyzed using the Illumina sequencing. A total of 12,879 MEAM1 transcripts and 11,246 MED transcripts were annotated with a significant Blastx hit. In addition, 7,000 and 5,771 gut specific genes were respectively identified for MEAM1 and MED. Functional analyses on these gut specific genes demonstrated the important roles of gut in metabolism of insecticides and secondary plant chemicals. To reveal the molecular difference between guts of MEAM1 and MED, a comparison between gut transcriptomes of the two species was conducted and 3,910 pairs of orthologous genes were identified. Based on the ratio of nonsynonymous and synonymous substitutions, 15 genes were found evolving under positive selection. Many of those genes are predicted to be involved in metabolism and insecticide resistance. Furthermore, many genes related to detoxification were expressed at an elevated level in the gut of MED compared to MEAM1, which might be responsible for the MED’s higher resistance to insecticides and environmental stresses.

**Conclusion:**

The sequencing of MED and MEAM1 gut transcriptomes and extensive comparisons of MEAM1 and MED gut transcripts provide substantial sequence information for revealing the role of gut in whiteflies.

**Electronic supplementary material:**

The online version of this article (doi:10.1186/1471-2164-15-370) contains supplementary material, which is available to authorized users.

## Background

Many hemipteran insects feed on phloem sap, which is composed of rich content of sucrose, relatively poor component of amino acids, and small quantities of proteins and inorganic substances [1]. The gut of these sap feeders is often supposed to have strong capacities in dealing with the unbalanced diet. Efficient absorption of limited organic nutrients, such as amino acids, high activity of phloem sugar hydrolysis, and maintenance of osmotic pressure at an appropriate level are speculated to be the main function of guts [2–4]. Compared to the sap of other plant cells, the fluid of phloem is a good diet with less toxic substances [5]. However, defense secondary metabolites and proteins, such as alkaloids, glucosinolates, glucosides, chitinase and protein inhibitors, are detectable in the phloem, and have been shown to exert negative effects on phloem feeders [6]. As a major organ of the insect digestive system, the gut is likely involved in detoxification of harmful substances in phloem during digestion and assimilation [7–9]. In addition, insect guts play important roles in pesticide resistance and xenobiotics metabolism [10–12]. Insecticide resistance can arise by over expression of detoxification enzymes such as cytochrome P450 monooxygenases (P450), UDP-glucuronosyltransferase (UGT), glutathione-S-transferases (GST) [13–17]. These proteins can convert insecticides and toxic compounds into less toxic or nontoxic chemicals [18]. Therefore, insect guts are critical in plant-insect interaction and insecticide resistance.

The whitefly *Bemisia tabaci* (Gennadius) (Hemiptera: Aleyrodidae) is now known as a complex of genetically distinct cryptic species that often differ in host range, insecticide resistance, capacity for virus transmission, and the symbionts they harbor [19–24]. Worldwide, more than 35 putative cryptic species of the complex have so far been identified [24–28]. *Bemisia tabaci* impairs plant by sucking phloem sap and transmitting over 100 species of plant viruses in the genus *Begomovirus* during feeding [29]. Within the species complex, the Mediterranean (MED, previously known as the Q ‘biotype’) and Middle East-Asia Minor 1 (MEAM1, previously known as the B ‘biotype’) species are highly invasive and have caused considerable economic damages to many important crops. MEAM1 invaded China in the mid-1990s, and displaced the native whiteflies of the *B. tabaci* complex rapidly and became the dominant whitefly species in many regions of invasion [19, 27]. In 2003, MED was first detected in Yunnan province of China [30], and since then has been rapidly spreading to many provinces and replacing other species in the *B. tabaci* complex including the earlier invader MEAM1 [27, 31–33]. Compared to some native whitefly species, many genes involved in drug metabolism and detoxification pathways were highly expressed in the invasive species, which may contribute to their invasion and displacement of other indigenous species [34]. In addition, several studies have revealed that the greater abundance of MED relative to MEAM1 in Israel and southern Spain were associated with its higher levels of resistance to pyriproxyfen and neonicotinoids [35, 36]. Comparison of MEAM1 and MED transcriptomes demonstrated that the sequence divergence of pesticide resistance genes may cause functional differences in corresponding enzymes and result in the biological variations [37]. However, no genomic information is yet available for the whitefly gut despite the importance of gut in plant-insect interaction and insecticide resistance,

Due to the fact that the body length of an adult whitefly is only *ca.* 1 mm and the size of its gut is much smaller, the collection of microgram amounts of total whitefly gut RNA is extremely difficult. To overcome this obstacle, we utilized the cDNA amplification method described in previous studies to obtain large amount of MEAM1 and MED gut nucleotide samples [34, 37, 38]. The amplified gut cDNA was used for library construction and Illumina sequencing. After sequence assembly, a total of 33,412 MEAM1 gut transcripts and 27,443 MED gut transcripts were obtained. Through the analysis of the transcriptome data, genomic features and putative functions of the whitefly gut were revealed. Furthermore, the divergences of MED and MEAM1 gut genes were analyzed and presented for the first time. This study provides a valuable source of molecular information for future functional studies on whitefly guts and will facilitate the research of guts in whitefly-plant interactions and insecticide resistance.

## Result and discussion

### Illumina sequencing, reads assembly and functional annotation

The amplified cDNA samples of MEAM1 and MED guts were separately sequenced using the Illumina HiSeq 2000 platform. Initially, about 30 and 29 million raw reads were obtained from the libraries of MEAM1 and MED guts, respectively (Table 1). The raw reads were filtered by removing those with adaptors and ambiguous nucleotides. After that, approximately 27 million clean reads were obtained for each sample. Subsequently, MEAM1 and MED gut transcriptomes were *de novo* assembled using the short reads assembling program – Trinity [39]. A total of 65,213 and 60,357 Inchworm contigs were assembled for MEAM1 and MED respectively. After paired-end joining and sequence clustering, 33,412 MEAM1 gut transcripts with the mean size of 625 nucleotides and 27,443 MED gut transcripts with the mean size of 632 nucleotides were acquired (Table 1). The lengths of these transcripts ranged from 300 to over 3,000 nucleotides. For functional annotation, all the transcripts of the two transcriptomes were searched against NCBI nr nucleotide database using BLASTx. For MEAM1 gut transcriptome, 12,879 transcripts got significant BLAST hits (E-value < 1.0E^−5^) (Additional file 1), and for MED gut, 11,246 transcripts got significant BLAST hits (E-value < 1.0E^−5^) (Additional file 2).

**Table 1:** **Summary for the MEAM1 and MED gut transcriptomes**

Features	MEAM1 gut	MED gut
Total number of raw reads	30,066,096	29,326,438
Total number of clean reads	27,222,864	26,782,986
Total clean nucleotides (nt)	2,450,057,760	2,410,468,740
Average read length (nt)	90	90
Total number of Inchworm contigs	65,213	60,357
Mean length of Inchworm contigs	354	330
Total transcripts	33,412	27,443
Mean length of transcript units (nt)	625	632

### Assignment of transcripts to Gene Ontology (GO) terms and Kyoto Encyclopedia of Genes and Genomes (KEGG) pathways

To further reveal their functions, GO assignments were used to classify the MED and MEAM1 gut transcripts. Based on sequence homology, a total of 7,127 MEAM1 and 6,305 MED gut sequences were categorized into 58 functional groups at level two under the ‘Biological process’, ‘Cellular component’ and ‘Molecular function’ divisions (Additional file 3). However, the profiles of the 58 GO groups in the two species had some differences. While ‘virion’ and ‘virion part’ were only present in the MEAM1 gut, ‘channel regulator activity’ and ‘carbohydrate utilization’ were unique to the MED gut. The top three groups of each division of these two transcriptomes were the same. Specifically, the top three groups in ‘Biological process’ were ‘cellular process’, ‘metabolic process’ and ‘biological regulation’; the top three groups in ‘Cellular component’ were ‘cell’, ‘cell part’ and ‘organelle’; and the top three groups in ‘Molecular function’ were ‘catalytic activity’, ‘binding’ and ‘transporter activity’.

**Figure 1:**
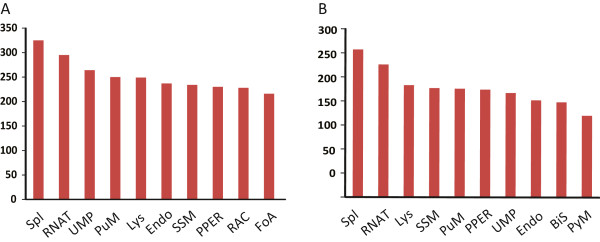
**Distribution of transcripts among the KEGG pathways. (A)** Transcripts from the MEAM1 gut. **(B)** Transcripts from the MED gut. The top ten pathways with highest numbers of transcripts mapped to were shown. Abbreviation for pathways: Spliceosome (Spl), RNA transportor (RNAT), Ubiquitin mediated proteolysis (UMP), Purine metabolism (PuM), Lysosome (Lys), Endocytosis (Endo), Starch and sucrose metabolism (SSM), Protein processing in endoplasmic reticulum (PPER), Regulation of actin cytoskeleton (RAC), Focal adhesion (FoA), Bile secretion (BiS), Pyrimidine metabolism (PyM).

In order to find out which biological pathways are active in whitefly guts, the MEAM1’s 33,412 transcripts and MED’s 27,443 transcripts were assigned to the reference pathways in KEGG. Consequently, 5,943 MEAM1 gut transcripts were mapped to 256 pathways and 5,254 MED gut transcripts were mapped to 253 pathways. Among these pathways, ‘Spliceosome’ (325), ‘RNA transport’ (295) and ‘Ubiquitin mediated proteolysis’ (264) (Figure 1A) were the most common pathways in MEAM1. As to the MED gut, ‘Spliceosome’ has the highest percentage of transcripts (307 transcripts), followed by ‘RNA transport’ (276 transcripts) and ‘Lysosome’ (233 transcripts) (Figure 1B).

### KEGG pathway enrichment analysis of gut genes

Enrichment analysis is an effective way to identify the KEGG pathways that frequently occur in a tissue with the whole body transcriptome as background [40, 41]. In MED, a total of 11 gut enriched KEGG pathways (P-value < 5.0E^−3^) were identified (Table 2). Whereas in MEAM1, there were 25 gut enriched pathways (Additional file 4). Even though the numbers of enriched pathways differ, the functions of the enriched pathways appeared similar between MED and MEAM1 guts. Pathways like ‘Metabolism of cofactors and vitamins’, ‘Carbohydrate metabolism’ and ‘Digestive system’ were enriched in both MEAM1 and MED guts. This is consistent with the principal function of sap-sucking insect’s gut – uptake of nutrients. In addition, ‘Membrane transport’ and ‘Transport and catabolism’ were also enriched. These transport-related pathways have been shown to be important during the secretion of digestive enzymes and the formation of gradient between the gut luminal sap and the perimicrovillar space [3, 42, 43]. Interestingly, ‘Xenobiotics biodegradation and metabolism’ pathway was highly enriched in MED, but not in MEAM1 (Table 2 and Additional file 4). In addition, pathways such as ‘Drug metabolism - other enzymes’, ‘Drug metabolism - cytochrome P450’, ‘Metabolism of xenobiotics by cytochrome P450’ were also enriched in MED gut (Table 2), which is consistent with the high insecticide resistance of MED whiteflies [44–47].

**Table 2:** **Statistically enriched KEGG pathways in MED guts**

KEGG	E-value	Gut genes^1^	WB genes^2^
Xenobiotics biodegradation and metabolism	0.00E + 00	465	518
Drug metabolism - cytochrome P450	6.61E-10	156	161
Metabolism of xenobiotics by cytochrome P450	1.18E-08	151	164
Drug metabolism - other enzymes	9.30E-07	158	193
Metabolism of cofactors and vitamins	4.18E-13	429	555
Retinol metabolism	3.97E-11	141	131
Porphyrin and chlorophyll metabolism	1.06E-06	124	141
Digestive system	8.97E-13	634	906
Bile secretion	0.00E + 00	198	117
Mineral absorption	8.40E-04	37	37
Fat digestion and absorption	2.18E-03	49	58
Carbohydrate digestion and absorption	4.32E-03	40	47
Transcription	1.99E-11	456	622
Spliceosome	7.64E-10	307	396
Carbohydrate metabolism	2.98E-07	1055	1793
Ascorbate and aldarate metabolism	1.47E-07	110	113
Pentose and glucuronate interconversions	2.68E-05	125	157
Membrane transport	5.67E-05	85	98
ABC transporters	5.67E-05	85	98
Excretory system	3.04E-04	204	305
Vasopressin-regulated water reabsorption	1.96E-04	79	94
Metabolism of Terpenoids and Polyketides	3.09E-04	66	76
Insect hormone biosynthesis	3.47E-04	29	24
Transport and catabolism	1.52E-03	683	1212
Lysosome	2.77E-03	233	377
Folding, sorting and degradation	2.16E-03	674	1201
Proteasome	7.76E-04	55	63
Lipid metabolism	4.01E-03	518	914
Steroid hormone biosynthesis	1.73E-07	124	134
Others			
Tight junction	1.52E-05	156	204
Cardiac muscle contraction	3.43E-03	77	105
Other types of O-glycan biosynthesis	3.30E-05	113	139
beta-Alanine metabolism	3.81E-03	37	42
Pyrimidine metabolism	3.14E-03	170	265
Phototransduction	1.71E-06	25	11
Olfactory transduction	1.26E-03	34	34
Ribosome biogenesis in eukaryotes	3.47E-04	150	213

^1^The number of gut genes in each of the KEGG pathways.

^2^The number of whole-body (WB) genes in each of the KEGG pathways.

### Gut specific genes

In order to reveal the specific function of whitefly guts, the orthologous genes between whitefly gut and whole body transcriptomes were identified using OrthoMCL [48]. The gut genes that cannot be classified into any orthologous groups were considered as gut specific genes. In our analysis, the MEAM1 gut specific genes were identified against the MEAM1 whole body transcriptome [37] and MED gut specific genes against the MED whole body transcriptome [49]. As a result, a total of 7,000 and 5,771 specific genes were identified for MEAM1 and MED guts respectively. The proportion of MEAM1 and MED gut specific genes to the whole gut transcriptome were nearly equal (20.95% and 21.03%). Next, these gut specific genes were classified through KEGG annotation (Table 3). The results showed that ‘Alpha-glucosidase’, ‘Facilitated trehalose transporter’ and ‘MFS transporter’ terms contained the most gut specific genes. This is consistent with the function of whitefly gut in sucrose hydrolysis and nutrient absorption. In addition, a number of detoxification-related genes such as cytochrome P450, GST and glucuronosyltransferase were also found specifically expressed in the gut (Table 3).

**Table 3:** **The KO classification of gut specific genes**

KO ID	Number of genes	KO definition
**MED gut**		
K00699	42	glucuronosyltransferase [EC:2.4.1.17]
K01363	35	cathepsin B [EC:3.4.22.1]
K01187	31	alpha-glucosidase [EC:3.2.1.20]
K09228	28	KRAB domain-containing zinc finger protein
K10955	27	intestinal mucin-2
K15002	23	cytochrome P450, family 6 [EC:1.14.-.-]
K14258	21	facilitated trehalose transporter
K14410	18	acid phosphatase [EC:3.1.3.2]
K00799	16	glutathione S-transferase [EC:2.5.1.18]
K01104	16	protein-tyrosine phosphatase [EC:3.1.3.48]
K08145	14	MFS transporter, SP family, solute carrier family 2
K03283	11	heat shock 70 kDa protein 1/8
K06115	11	spectrin beta
K10380	11	ankyrin
K14972	11	PAX-interacting protein 1
K05658	10	ATP-binding cassette, subfamily B (MDR/TAP)
**MEAM1 gut**		
K01187	39	alpha-glucosidase [EC:3.2.1.20]
K09228	33	KRAB domain-containing zinc finger protein
K00699	22	glucuronosyltransferase [EC:2.4.1.17]
K15002	22	cytochrome P450, family 6 [EC:1.14]
K01363	21	cathepsin B [EC:3.4.22.1]
K14258	21	facilitated trehalose transporter
K10955	18	intestinal mucin-2
K00799	15	glutathione S-transferase [EC:2.5.1.18]
K01104	14	protein-tyrosine phosphatase [EC:3.1.3.48]
K08145	13	MFS transporter, SP family, solute carrier family 2
K10380	13	ankyrin
K05643	11	ATP-binding cassette, subfamily A (ABC1), member 3
K08052	11	neurofibromin 1
K11789	11	HIV-1 Vpr-binding protein
K10382	10	dystonin
K10594	10	E3 ubiquitin-protein ligase HERC1 [EC:6.3.2.19]
K14209	10	solute carrier family 36 (proton-coupled amino acid transporter)

Note: ‘The KO IDs with more than 10 gut genes were shown in this table’.

**Figure 2:**
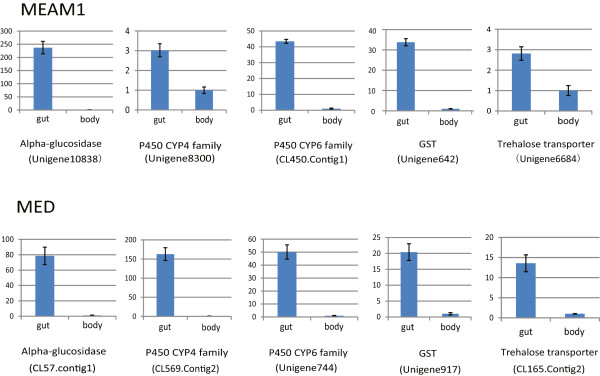
**Expression of gut specific genes.** In each assay, the expression level was normalized to the lowest expression level, which was arbitrarily set at one. The number in parentheses represents the ID number of transcripts in the MEAM1 and MED gut transcriptomes.

To validate whether these gut genes are specifically expressed, several genes were randomly picked out to analyze their expression levels. Total RNA was separately extracted from the gut and the rest of the body of MEAM1 and MED whiteflies. Quantitative real-time PCR (qPCR) results showed that all the selected genes were highly or specifically expressed in the gut (Figure 2). The expression of these genes was significantly low or almost non-detectable in the rest of whitefly body.

### Orthologous genes between the MED and MEAM1 gut transcriptomes

To compare the sequence divergence of the MEAM1 and MED gut genes, a bidirectional best hit approach was used to identify orthologous genes [37, 50, 51]. By this way, 14,472 pairs of putative orthologous genes between MEAM1 and MED guts were identified (Additional file 5). To filter out potential paralogs, only pairs of sequences mapped unambiguously to the same protein in Swissprot database with an E-value < 1E^−5^ were selected as orthologous genes. As a result, 3,987 pairs of orthologous genes were identified between MEAM1 and MED gut transcriptomes (Additional file 5). The coding sequence (CDS) and untranslated region (UTR) of each sequence pair were identified based on the predicted coding region. After filtering the sequences shorter than 75 bp, a total of 3,910 pairs of orthologous CDS were identified (Additional file 5 and Additional file 6). The average length of the 3,910 orthologous genes was 677.7 bp with an average similarity of 99.25%.

### The sequence divergence between MEAM1 gut and MED gut orthologous genes

The sequence divergence of orthologous genes between MEAM1 and MED guts was analyzed to reveal their molecular variation. As shown in Table 4, the overall difference between 5’UTRs of MEAM1 and MED gut orthologous genes was 1.69% and the difference between 3’UTR of MEAM1 and MED orthologous genes was 1.59%. When it comes to the CDS, the overall divergence among the 3,910 orthologous gene pairs was 0.75%. The lower divergence rate at CDS might be due to the high selection pressure. In coding regions, the nucleotides can be further classified as nondegenerative (nd) sites (any substitutions result in amino acid change) and four fold degenerate (4d) sites (no changes produce amino acid replacement). From a total of 2,649.92 kb of CDS, a total of 1,551.93 kb were nd sites, whereas 375.10 kb were 4d sites (Table 4). As any nucleotide substitutions at nd sites will produce amino acid changes, the nd sites are under extensive functional constraints in the evolution process. Indeed, at 4d sites, the divergence between MEAM1 and MED was 2.48%, 14.6 times of that at nd sites (0.17%) (Table 4). Therefore, the difference at 4d sites might be the main source of sequence divergence between MEAM1 and MED gut genes. In order to verify the sequence divergence, 5 pairs of genes with known point mutations were randomly selected for cloning and Sanger sequencing from both MEAM1 and MED guts (Additional file 7). Our results showed that: i) for both species, the sequences from the *de novo* assembled transcriptome and Sanger sequencing are identical; ii) all of the differences between MEAM1 and MED revealed from the bioinformatic analysis were confirmed with Sanger sequencing. These results suggest that the transcriptome sequences of MEAM1 and MED guts and their divergence are reliable (Additional file 7).

**Table 4:** **Sequence divergence between MED and MEAM1 gut transcriptomes**

			% Differences		
	%GC	Loci	Mean	SE	Compared kb
5’UTRs^a^	39.28	685	1.69	0.09	109.69
CDS^b^	43.46	3910	0.75	0.01	2649.92
nd sites^c^	44.21	3910	0.17	0.01	1551.93
4d sites^d^	37.74	3910	2.48	0.05	375.10
3’ UTRs	32.79	1013	1.59	0.07	336.37

^a^UTRs: untranslated regions.

^b^CDS: coding sequence.

^c^nd sites: non-degenerative sites.

^d^4d sites: fourfold-degenerate sites where no changes cause any amino acid replacement.

### Analysis of sequences with weak amino-acid similarity

The 3,910 sequence pairs between MEAM1 and MED guts had a mean homology of 99.25% and ranged from 78.38% to 100% (Additional file 6). The functions of sequence pairs with weak amino-acid similarity were analyzed to reveal the proteins responsible for the differences between the two species. Many of the highly diverged genes were related to sugar metabolism such as gene Pair 2223 (sequence identity: 93.69%) and gene Pair 3793 (sequence identity: 95.30%), both of which encode alpha-glucosidase (Additional file 6). In addition, gene Pair 3748 encoding a sugar transporter also showed high sequence divergence (sequence identity: 96.12%). We also noticed that genes related to ‘Xenobiotics metabolism’ were highly diverged, such as carboxylesterase clade E (95.13%), two GSTs (95.19%, 96.26%) and two UGTs (95.45%, 95.56%) (Additional file 6).

Furthermore, the 3,910 MEAM1 and MED gut orthologous genes were classified by KEGG pathways and the average divergences of genes in each pathway were calculated. Interestingly, the most divergent pathways were those related to metabolism, such as ‘Steroid hormone biosynthesis’, ‘Drug metabolism - cytochrome P450’ and ‘Metabolism of xenobiotics by cytochrome P450’ (Table 5). These data indicate that these pathways of xenobiotics biodegradation and metabolism may result in the difference of some major biological characteristics such as insecticide resistance. Besides, ‘Starch and sucrose metabolism’ and ‘Ascorbate and aldarate metabolism’ also had a low average identity, suggesting that MEAM1 and MED guts may have different metabolic capacities as well. Furthermore, the high divergence of genes in ‘Nicotinate and nicotinamide metabolism’ pathway might be responsible for the differences in host plant utilization between MEAM1 and MED whiteflies (Table 5).

**Table 5:** **Average identities of orthologous genes between MEAM1 and MED guts in different KEGG pathways**

Pathway ID	Pathway description	Number of gene pairs	Average identity
ko00140	Steroid hormone biosynthesis	30	0.9767
ko00982	Drug metabolism - cytochrome P450	38	0.9770
ko00053	Ascorbate and aldarate metabolism	25	0.9777
ko00980	Metabolism of xenobiotics by cytochrome P450	37	0.9778
ko00040	Pentose and glucuronate interconversions	30	0.9790
ko00514	Other types of O-glycan biosynthesis	30	0.9806
ko00983	Drug metabolism - other enzymes	41	0.9811
ko00860	Porphyrin and chlorophyll metabolism	36	0.9816
ko00830	Retinol metabolism	38	0.9819
ko00760	Nicotinate and nicotinamide metabolism	11	0.9827
ko00350	Tyrosine metabolism	13	0.9830
ko00500	Starch and sucrose metabolism	71	0.9833
ko00563	Glycosylphosphatidylinositol(GPI)-anchor biosynthesis	13	0.9846
ko04974	Protein digestion and absorption	26	0.9850
ko04976	Bile secretion	52	0.9853

### Synonymous and nonsynonymous sites

**Figure 3:**
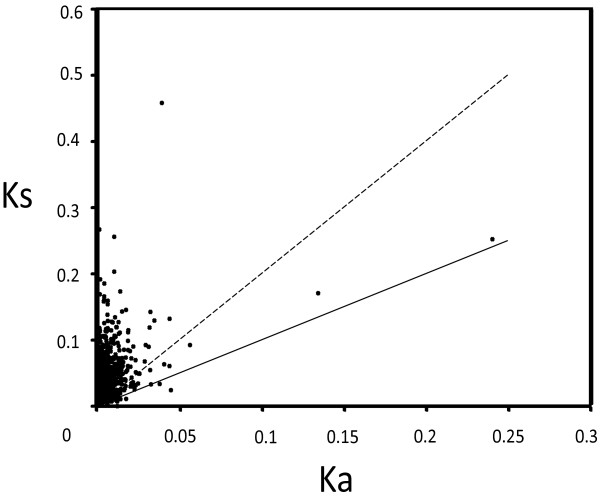
**Distribution of Ka and Ks.** Sequences with Ka/Ks > 1 fall below the solid line; while sequences with Ka/Ks between 0.5 -1 fall between the solid and dashed lines.

To classify genes undergoing purifying and positive selections, the substitution rates of synonymous (Ks) and nonsynonymous (Ka) sites between MEAM1 and MED gut ortholog pairs were calculated. A total of 1,080 ortholog pairs generated both Ka and Ks (Figure 3 and Additional file 8). The mean value of Ka of these 1,080 sequences was 0.0056 and the mean value of Ks was 0.0392. The average Ka/Ks ratio was 0.1951, which is similar to the average Ka/Ks ratio of MEAM1 and MED whole body (0.225) [37]. In the 1,080 selected orthologous gene pairs, the majority of genes’ Ka/Ks ratios were less than 1 (98.61%, 1065/1080), indicating purifying selection for these genes [52]. At the same time, 15 gene pairs’ Ka/Ks values were larger than 1 (Table 6), indicating that these genes are under strong positive selection. Some of the genes are related to energy metabolism and xenobiotic metabolism, such as NADH dehydrogenase, ATP synthase, mannosyl-oligosaccharide 1 and UGT, suggesting that these processes may be critical to the difference between MEAM1 and MED guts.

**Table 6:** **List of gene pairs with Ka/Ks larger than one**

Gene pair ID	S-Sub^a^	N-Sub^b^	Ka^c^	Ks^d^	Ka/Ks	Protein homolog
1245	1	6	0.0062	0.0032	1.9268	ER degradation-enhancing alpha-mannosidase
2260	2	6	0.0451	0.0241	1.8694	Paternally-expressed gene 3 protein
1570	1	6	0.0124	0.0070	1.7688	ATP synthase subunit s-like protein
3878	2	12	0.0178	0.0109	1.6398	Eukaryotic translation initiation factor
2569	1	4	0.0065	0.0040	1.5995	Solute carrier family 25 member 38
1312	1	4	0.0143	0.0105	1.3721	NADH dehydrogenase
1207	1	5	0.0117	0.0088	1.3270	NADH dehydrogenase
911	1	4	0.0089	0.0072	1.2397	Uncharacterized protein
1382	2	5	0.0091	0.0076	1.2037	UDP-glucuronosyltransferase
3874	2	6	0.0383	0.0335	1.1414	Steroid 17-alpha-hydroxylase/17,20 lyase
91	1	3	0.0079	0.0075	1.0525	Uncharacterized protein
385	1	3	0.0061	0.0058	1.0478	Uncharacterized protein
2769	3	9	0.0127	0.0125	1.0144	39S ribosomal protein
2661	1	3	0.0076	0.0075	1.0142	28S ribosomal protein
3823	6	14	0.0329	0.0328	1.0037	Glutamic acid-rich protein

^a^S-Sub Synonymous substitutions.

^b^N-Sub Nonsynonymous substitutions.

^c^Ka Nonsynonymous substitution rate.

^d^Ks Synonymous substitution rate.

### Differential expression of the orthologous genes between MEAM1 and MED guts

To compare the level of gene expression between MEAM1 and MED gut orthologous genes, the number of reads mapped to each orthologous gene pairs was extracted. Among the 3,910 orthologous genes between MEAM1 and MED guts, a total of 64 genes were down-regulated and 304 were up-regulated in MEAM1 (log2 > 1, FDR value < 1.0E^−3^) (Additional file 9). In order to validate the data from bioinformatic analysis, the gene expression between 20 MEAM1 and MED gut genes were examined using qPCR (10 MEAM1 up-regulated genes and 10 MEAM1 down-regulated genes). Out of the 20 selected genes, 19 showed a concordant direction of change between transcriptome analysis and qPCR quantification results, which confirmed the reliability of our analyses (Additional file 10).

**Figure 4:**
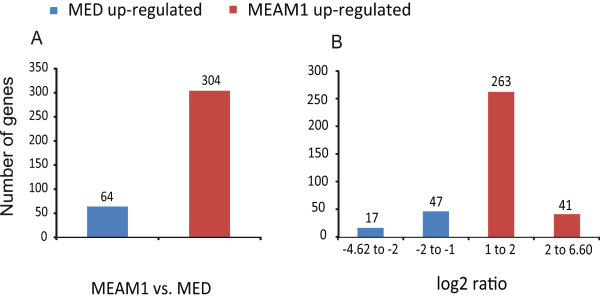
**Differential expressions of the orthologous genes between MEAM1 and MED guts. (A)** The numbers of differentially expressed genes between MEAM1 and MED guts. **(B)** The log_2_ ratio distribution of differentially expressed genes between MEAM1 and MED gut transcriptomes.

Of the differentially expressed genes, the detected fold changes (log2 ratio) of gene expression ranged from minimum −4.62 to maximum 6.60 (Figure 4). The majority of orthologous genes were up- or down-regulated between 1.0 and 2.0 fold, whereas 41 orthologous pairs were with a value of log2 > 2, and 17 orthologous pairs with a value of log2 < −2 (Figure 4). Among all these 58 differentially expressed gene pairs, 4 detoxification-related genes (3 P450s and 1 GST) were significantly up-regulated in MED gut compared with MEAM1 gut, and none of them was up-regulated in MEAM1, which may be responsible for the difference in insecticide resistance between MEAM1 and MED.

## Conclusions

In this study, we sequenced the transcriptomes of MEAM1 and MED guts, and obtained 33,412 MEAM1 and 27,443 MED gut transcripts. Among these genes, 12,879 MEAM1 and 11,246 MED transcripts had significant nr BLAST hits. The main function of whitefly gut was revealed by the GO and KEGG analysis of MEAM1 and MED transcriptomes. Then, 3,910 orthologous genes pairs between MED and MEAM1 guts were identified. Gene divergence analysis showed that 15 genes were under strong positive selection and many of them are related to energy and xenobiotic metabolism. In addition, some of the drug metabolism and detoxification related genes such as P450s, GSTs were expressed at a higher level in MED than in MEAM1. The higher expression and divergence of these genes may contribute to the high detoxification ability and insecticide resistance of MED. To our knowledge, this is the first substantial sequencing and comparison of gut transcriptomes in whiteflies. These analyses provide a valuable resource of molecular information for future investigations of the functions of whitefly gut genes.

## Methods

### Whitefly rearing, sample collection and RNA isolation

Cotton *Gossypium hirsutum* L. (Malvaceae) cv. Zhe-Mian 1793 was used as the host plant for rearing MEAM1 and MED whiteflies. Whiteflies were maintained in climate chambers at 27 ± 1 °C, a photoperiod of 14 h/10 h and 70 ± 10% relative humidity. Every 3–5 generations, the purity of the cultures was monitored using the random amplified polymorphic DNA polymerase chain reaction technique with the primer H16 (5’-TCTCAGCTGG-3’), which has been used widely to distinguish different species in the *B. tabaci* complex [53], and further confirmed by mtCOI sequencing of a few individuals. The cultures of the two species were raised from a pair of virgin adult whiteflies of MEAM1 or MED and maintained on cotton for 3 generations before they were taken for experiments. For sample collection and RNA isolation, about 200 guts were dissected from the MEAM1 and MED adult whiteflies in PBS respectively. Then total gut RNA was isolated using the Absolutely RNA Nanoprep kit (Agilent, USA) according to the manufacturer’s manual with slight modifications [38].

### Sequencing library construction

The gut cDNA was prepared by using a SMARTer™ PCR cDNA Synthesis Kit (Clontech, USA) and an Advantage 2 PCR Kit (Clontech, USA) as described previously [38]. Briefly, the RNA sample was reverse transcribed to first-strand cDNA. Then, the first-strand cDNA product was used for PCR amplification. To determine the optimal number of PCR cycles, 5 μl of PCR products at 15, 18, 21, 24, 27 and 30 cycles were checked by agarose gel electrophoresis. According to the analysis, 2 μl of first-strand cDNA was amplified for 26 cycles. Then, the amplified cDNA was purified by QIAquick PCR purification kit (Qiagen, Germany). At last, library for transcriptome sequencing was prepared using Illumina kit following the manufacturer’s recommendations.

### Illumina sequencing and transcript annotation

The MEAM1 and MED gut libraries were sequenced for both ends using Illumina HiSeq 2000 platform at Beijing Genomics Institute (Shenzhen, China). The total sequencing amount was 3G for each library. After removing the low quality reads, *de novo* transcriptome assembly was carried out with the Trinity program [39]. There are three independent modules in the Trinity program, known as “Inchworm”, “Chrysalis” and “Butterfly”. The module “Inchworm” assembled the RNA-Seq data into unique transcripts which we called Inchworm contigs. “Chrysalis” clustered the Inchworm contigs and constructed complete de Bruijn graphs for each cluster and then partitioned the full read set among these disjoint graphs. “Butterfly” processed the individual graphs in parallel, tracing the paths based on reads and pair-end information, ultimately reporting full-length transcripts for alternatively spliced isoforms. After Trinity assembly, TGICL with parameter setting “-l 40 -c 10” was used to cluster and remove redundant transcripts. The remaining sequences after TGICL clustering were defined as tentative transcripts. The transcripts can be divided into two classes. One is cluster, which include several transcripts with more than 70% similarity among them and the prefix is CL. The other class is singletons with the prefix unigene. Next, Blastx search (E-value < 1E^−5^) against the NCBI nr, Swiss-Prot and KEGG databases was performed and the best aligning results were used to decide the direction of transcripts. If results of different databases conflict with each other, a priority order of nr, SwissProt and KEGG were followed to decide the direction of transcripts. The data sets are available at the NCBI Short Read Archive (SRA) with the accession number: SRR835757 (MEAM1 gut) and SRR835756 (MED gut). The assembled sequences have been deposited in the NCBI's TSA database: GAPP00000000 (MEAM1 gut) and GAPQ00000000 (MED gut).

### Identification of statistically enriched pathways

KEGG enrichment analysis is an effective way to identify the enriched pathways in a specific tissue using the whole body transcriptome as a background [54]. The significantly enriched pathways in the gut were identified by a hypergeometric test with the calculating formula: . In this formula, N and n represent the total numbers of whole body and gut transcriptome genes with KEGG annotations, while M, m are the numbers of whole body and gut transcriptome genes annotated to a certain KEGG pathway. The pathways with a *p*-value less than 5.0E^−3^ were determined as enriched. In our analyses, human diseases associated pathways were excluded.

### Identification of gut specific expressed genes

The OrthoMCL program was used to detect MEAM1 and MED gut specific genes compared to the whole body transcriptomes of MEAM1 and MED [48]. Complete lists (in FASTA format) of all predicted proteins in gut and whole body transcriptomes were used as templates. The OrthoMCL program began with all-vs-all BLASTP of the complete protein set [55]. Putative orthologous relationships were identified between gut and body by reciprocal best similarity pairs. The OrthoMCL program classified all putative orthologous into orthologous groups. Thus, gut genes that could not be categorized into any of the orthologous groups were regarded as gut specifically expressed genes.

### Orthologous genes between MEAM1 and MED gut transcriptomes

The orthologous genes between MEAM1 gut and MED gut transcriptomes were identified by MegaBLAST [37, 50]. Only pairs of sequences that were best hit (E-value < 1E^−5^) to each other and longer than 200 bp were retained as putative orthologs. To prevent potential orthologous paralogs, all putative orthologs obtained in the previous step were further filtered and only pairs of sequences unambiguously mapped to the same protein in Swissprot database with an E-value < 1E^−5^ were selected [34]. Transcripts were firstly aligned by Blastx (E-value < 1.0E^−5^) to nr, Swiss-Prot and KEGG databases. Proteins with the highest rank in Blast results were taken to identify the CDS of transcripts. CDS with unexpected stop codon in the Blast hit region and shorter than 75 bp were removed. The 5’UTR were defined based on the position of start codon and 3’UTR regions were defined based on the position of stop codon and predicted CDS.

### Sequence divergence analysis and estimation of substitution rates

The 5’UTR, CDS and 3’UTR regions were separately extracted from each pair of orthologs. To ensure the sequence comparison was performed only at the homologous regions of each gene pair, CDS and UTRs regions were aligned to each other separately and checked manually for errors. Sequence divergences between the homologous regions of each gene pair were calculated by dividing the number of substitutions with the number of base pairs in the comparison. The divergence was determined for both nd and 4d sites. In addition, Ka and Ks were estimated using the KaKs Calculator (YN method) [56, 57].

### Differential expression of orthologous genes

To compare the level of gene expression between MEAM1 and MED guts, the number of reads mapped to each orthologous gene pairs was extracted. Since read mapping is sensitive to the size of the target reference sequence and sequencing amount, the raw read counts were adjusted by the total number of reads mapped and the length of the gene by calculating Reads Per Kilo-base per Million mapped reads (RPKM). The fold change of the gene expression level of ortholog gene pairs in MEAM1 and MED transcriptomes were calculated with log _2_ (RPKM of the MEAM1 gut gene/RPKM of the MED gut gene). After that, significance of gene expression differences was tested using the algorithm established by Audic *et al.*[58]. At last the Benjamini Hochberg procedure was used for multiple testing correction and estimating the False Discovery Rate (FDR) [59].

### Real-time quantitative PCR analysis

To validate gut specific expressed genes, total RNA was extracted from gut and the rest of the body (excluding gut) respectively. Then, all samples were used for first-strand cDNA synthesis with a PrimeScript RT reagent kit (Takara, Japan). Amplification of those cDNA samples was carried out in Bio-Rad CFX96TM Real-Time System (Bio-Rad, USA) using SYBR Premix Ex Taq TM II (Takara, Japan). The relative expression levels of each gene in gut and body were normalized using the threshold cycle (Ct) values that were obtained from reactions run on the same plate. TATA Box Binding Protein-Associated Factor (TAF) was chosen as the endogenous reference gene in qPCR analysis with the 2^-△△Ct^ method [60, 61]. To confirm the results of RPKM analyses of orthologous gene expression between MEAM1 and MED gut, the levels of expression of 20 selected genes (10 MEAM1 up-regulated genes and 10 MEAM1 down-regulated genes) were measured. The primers of these genes were designed based on the perfectly matched orthologous region (Additional file 10).

## Electronic supplementary material

Additional file 1: **Top BLAST hits of MEAM1 gut transcripts.** BLAST results against the NCBI nr database for all the transcripts with a cut-off E-value <1.0E^−5^ are shown. (XLSX 843 KB)

Additional file 2: **Top BLAST hits of MED gut transcripts.** BLAST results against the NCBI nr database for all the transcripts with a cut-off E-value <1.0E^−5^ are shown. (XLSX 745 KB)

Additional file 3: **Histogram presentation of GO classification of genes from the MEAM1 and MED gut transcriptomes.** The results are summarized in three main categories “Biological process”, “Cellular component” and “Molecular function”. The right y-axis indicates the number of genes in a category and the left y-axis indicates the percentage of a specific category of genes in that main category. (PDF 2 MB)

Additional file 4: **MEAM1 guts enriched KEGG pathways.** The MEAM1 gut enriched pathways (level 2) was identified by a hypergeometric test with MEAM1 whole body transcriptome as the background. Pathways with E-value <5.0E^−3^ were regarded as enriched. (XLSX 10 KB)

Additional file 5: **Identification and analysis of the orthologous genes between the gut transcriptomes of MEAM1 and MED.** The orthologous genes were identified by bidirectional best hit method using MegaBLAST. All putative orthologs were further filtered against the Swissprot database. The putative orthologs that hit to different genes in the Swissprot database were removed. The CDS of the orthologous genes were determined by BLASTx against the Swissprot database with a threshold E-value of 1.0E^−5^. After removing the UTR regions, sequences shorter than 75 bp and with unexpected codons in the CDS were filtered. (PDF 314 KB)

Additional file 6: **List of the orthologous gene pairs between MEAM1 and MED gut transcripts.** The length of orthologous region, identity and Nr annotations are shown. (XLSX 443 KB)

Additional file 7: **Verification of the sequence divergence between MEAM and MED.** Five pairs of orthologous genes that are different in sequence between MEAM1 and MED were cloned and sequenced. The Transcript ID and primer sequences are listed in the table. (XLSX 9 KB)

Additional file 8: **Ka and Ks of each orthologous gene pairs.** S-Substitutions: synonymous substitutions; N-Substitutions: nonsynonymous substitutions; Ka: nonsynonymous substitution rate; Ks: synonymous substitution rate. Nr ID and Nr annotation are also shown. (XLSX 154 KB)

Additional file 9: **Differentially expressed orthologous genes between MEAM1 and MED guts.** The differentially expressed orthologous genes (overlapping region ≥ 200 bp, FDR-value <E^−3^ and absolute value of fold change ≥ 2) are shown. RPKM, FDR-value, fold change (log_2_ ratio) of gene expression and best hit to nr KEGG and Swissprot database (E-value <1.0E^−5^) for all the gene pairs are also listed in this table. Nr annotations are also shown. (XLSX 77 KB)

Additional file 10: **Real time quantitative PCR (qPCR) analysis.** Twenty orthologous genes were selected for validation of expression level between MEAM1 and MED gut using qPCR. In this table, we listed the fold change values revealed by the transcriptome analysis, the fold change obtained by RT-PCR, and the primers for the RT-PCR. (XLSX 12 KB)

## References

[1] Ziegler H, Zimmermann MH, Milburn JA (1975). Nature of transported substances. Transport in Plants I.

[2] Douglas AE (2013). The nutritional physiology of aphids. Adv Insect Physiol.

[3] Terra WR (1990). Evolution of digestive systems of insects. Annu Rev of Entomol.

[4] Cristofoletti PT, Ribeiro AF, Deraison C, Rahbe Y, Terra WR (2003). Midgut adaptation and digestive enzyme distribution in a phloem feeding insect, the pea aphid *Acyrthosiphon pisum*. J Insect Physiol.

[5] Douglas AE (2006). Phloem-sap feeding by animals: problems and solutions. J Exp Bot.

[6] Walz C, Giavalisco P, Schad M, Juenger M, Klose J, Kehr J (2004). Proteomics of curcurbit phloem exudate reveals a network of defence proteins. Phytochemistry.

[7] Parde VD, Sharma HC, Kachole MS (2010). *In vivo* inhibition of *Helicoverpa armigera* gut pro-proteinase activation by non-host plant protease inhibitors. J Insect Physiol.

[8] Bolter C, Jongsma MA (1997). The adaptation of insects to plant protease inhibitors. J Insect Physiol.

[9] Broadway RM, Duffey SS (1986). Plant proteinase inhibitors: Mechanism of action and effect on the growth and digestive physiology of larval *Heliothis zea* and *Spodoptera exiqua*. J Insect Physiol.

[10] Matthews HJ, Down RE, Audsley N (2010). Effects of *Manduca sexta* allatostatin and an analogue on the peach-potato aphid *Myzus persicae* (hemiptera: aphididae) and degradation by enzymes in the aphid gut. Arch Insect Biochem Physiol.

[11] Candas M, Loseva O, Oppert B, Kosaraju P, Bulla LA (2003). Insect resistance to *Bacillus thuringiensis*: alterations in the indianmeal moth larval gut proteome. Mol Cell Proteomics.

[12] Down RE, Matthews HJ, Audsley N (2011). Oral activity of FMRFamide-related peptides on the pea aphid *Acyrthosiphon pisum* (Hemiptera: Aphididae) and degradation by enzymes from the aphid gut. Regul Pept.

[13] Ranson H, Claudianos C, Ortelli F, Abgrall C, Hemingway J, Sharakhova MV, Unger MF, Collins FH, Feyereisen R (2002). Evolution of supergene families associated with insecticide resistance. Science.

[14] Jones RT, Bakker SE, Stone D, Shuttleworth SN, Boundy S, McCart C, Daborn PJ, ffrench-Constant RH, van den Elsen JM (2010). Homology modelling of *Drosophila* cytochrome P450 enzymes associated with insecticide resistance. Pest Manag Sci.

[15] Puinean AM, Foster SP, Oliphant L, Denholm I, Field LM, Millar NS, Williamson MS, Bass C (2010). Amplification of a cytochrome P450 gene is associated with resistance to neonicotinoid insecticides in the aphid *Myzus persicae*. PLoS Genet.

[16] Enayati AA, Ranson H, Hemingway J (2005). Insect glutathione transferases and insecticide resistance. Insect Mol Biol.

[17] Despres L, David JP, Gallet C (2007). The evolutionary ecology of insect resistance to plant chemicals. Trends Ecol Evol.

[18] Yan L, Yang P, Jiang F, Cui N, Ma E, Qiao C, Cui F (2012). Transcriptomic and phylogenetic analysis of *Culex pipiens quinquefasciatus* for three detoxification gene families. BMC Genomics.

[19] Jiu M, Zhou XP, Tong L, Xu J, Yang X, Wan FH, Liu SS (2007). Vector-Virus mutualism accelerates population Increase of an invasive whitefly. PLoS One.

[20] Crowder DW, Horowitz AR, De Barro PJ, Liu SS, Showalter AM, Kontsedalov S, Khasdan V, Shargal A, Liu J, Carriere Y (2010). Mating behaviour, life history and adaptation to insecticides determine species exclusion between whiteflies. J Anim Ecol.

[21] Gorman K, Slater R, Blande JD, Clarke A, Wren J, McCaffery A, Denholm I (2010). Cross-resistance relationships between neonicotinoids and pymetrozine in *Bemisia tabaci* (Hemiptera: Aleyrodidae). Pest Manag Sci.

[22] Ghanim M, Morin S, Czosnek H (2001). Rate of *Tomato yellow leaf curl virus* translocation in the circulative transmission pathway of its vector, the whitefly *Bemisia tabaci*. Phytopathology.

[23] Bing XL, Yang J, Zchori-Fein E, Wang XW, Liu SS (2013). Characterization of a newly discovered symbiont of the whitefly *Bemisia tabaci* (Hemiptera: Aleyrodidae). Appl Environ Microbiol.

[24] De Barro PJ, Liu SS, Boykin LM, Dinsdale AB (2011). *Bemisia tabaci*: A statement of species status. Ann Rev Entomol.

[25] Dinsdale A, Cook L, Riginos C, Buckley YM, De Barro P (2010). Refined global analysis of *Bemisia tabaci* (Hemiptera: Sternorrhyncha: Aleyrodoidea: Aleyrodidae) mitochondrial cytochrome oxidase 1 toidentify species level genetic boundaries. Ann Enomol Soc Am.

[26] Boykin LM, Shatters RG, Rosell RC, McKenzie CL, Bagnall RA, De Barro P, Frohlich DR (2007). Global relationships of *Bemisia tabaci* (Hemiptera: Aleyrodidae) revealed using Bayesian analysis of mitochondrial COI DNA sequences. Mol Phylogenet Evol.

[27] Hu J, De Barro P, Zhao H, Wang J, Nardi F, Liu SS (2011). An extensivefield survey combined with a phylogenetic analysis reveals rapid and widespread invasion of two alien whiteflies in China. PLoS One.

[28] Firdaus S, Vosman B, Hidayati N, Supena J, Darmo E, Visser RGF, van Heusden AW (2013). The *Bemisia tabaci* species complex: Additions from different parts of the world. Insect Sci.

[29] Hogenhout SA, Ammar ED, Whitfield AE, Redinbaugh MG (2008). Insect vector interactions with persistently transmitted viruses. Annu Rev Phytopathol.

[30] Chu D, Zhang YJ, Brown JK, Cong B, Xu BY, Wu QJ, Zhu GR (2006). The introduction of the exotic Q biotype of *Bemisia tabaci* from the Mediterranean region into China on ornamental crops. Florida Entomologist.

[31] Chu D, Wan FH, Zhang YJ, Brown JK (2010). Change in the biotype composition of *Bemisia tabaci* in Shandong Province of China from 2005 to 2008. Environ Entomol.

[32] Pan H, Chu D, Ge D, Wang S, Wu Q, Xie W, Jiao X, Liu B, Yang X, Yang N, Su Q, Xu B, Zhang Y (2011). Further spread of and domination by *Bemisia tabaci* (Hemiptera: Aleyrodidae) biotype Q on field crops in China. J Econ Entomol.

[33] Sun DB, Liu YQ, Qin L, Xu J, Li FF, Liu SS (2013). Competitive displacement between two invasive whiteflies: insecticide application and host plant effects. Bull Entomol Res.

[34] Wang XW, Zhao QY, Luan JB, Wang YJ, Yan GH, Liu SS (2012). Analysis of a native whitefly transcriptome and its sequence divergence with two invasive whitefly species. BMC Genomics.

[35] Horowitz AR, Denholm I, Gorman K, Cenis JL, Kontsedalov S, Ishaaya I (2003). Biotype Q of *Bemisia tabaci* identified in Israel. Phytoparasitica.

[36] Fernandez E, Gravalos C, Haro PJ, Cifuentes D, Bielza P (2009). Insecticide resistance status of *Bemisia tabaci* Q-biotype in south-eastern Spain. Pest Manag Sci.

[37] Wang XW, Luan JB, Li JM, Su YL, Xia J, Liu SS (2011). Transcriptome analysis and comparison reveal divergence between two invasive whitefly cryptic species. BMC Genomics.

[38] Su YL, Li JM, Li M, Luan JB, Ye XD, Wang XW, Liu SS (2012). Transcriptomic analysis of the salivary glands of an invasive whitefly. PLoS One.

[39] Grabherr MG, Haas BJ, Yassour M, Levin JZ, Thompson DA, Amit I, Adiconis X, Fan L, Raychowdhury R, Zeng Q, Chen Z, Mauceli E, Hacohen N, Gnirke A, Rhind N, Palma F, Birren B, Nusbaum C, Lindblad-Toh K, Friedman N, Regev A (2011). Full-length transcriptome assembly from RNA-Seq data without a reference genome. Nat Biotechnol.

[40] Mao X, Cai T, Olyarchuk JG, Wei L (2005). Automated genome annotation and pathway identification using the KEGG Orthology (KO) as a controlled vocabulary. Bioinformatics.

[41] Fukushima A, Kusano M, Redestig H, Arita M, Saito K (2011). Metabolomic correlation-network modules in Arabidopsis based on a graph-clustering approach. BMC Syst Biol.

[42] Lehane MJ, Blakemore D, Williams S, Moffatt MR (1995). Regulation of digestive enzyme levels in insects. Comp Biochem Physiol B Biochem Mol Biol.

[43] Naikkhwah W, O'Donnell MJ (2012). Phenotypic plasticity in response to dietary salt stress: Na + and K + transport by the gut of *Drosophila melanogaster* larvae. J Exp Biol.

[44] Yuan L, Wang S, Zhou J, Du Y, Zhang Y, Wang J (2012). Status of insecticide resistance and associated mutations in Q-biotype of whitefly, *Bemisia tabaci*, from eastern China. Crop Prot.

[45] Ghanim M, Kontsedalov S (2007). Gene expression in pyriproxyfen-resistant *Bemisia tabaci* Q biotype. Pest Manag Sci.

[46] Horowitz AR, Kontsedalov S, Khasdan V, Ishaaya I (2005). Biotypes B and Q of *Bemisia tabaci* and their relevance to neonicotinoid and pyriproxyfen resistance. Arch Insect Biochem.

[47] Nauen R, Stumpf N, Elbert A (2002). Toxicological and mechanistic studies on neonicotinoid cross resistance in Q-type *Bemisia tabaci* (Hemiptera: Aleyrodidae). Pest Manag Sci.

[48] Li L, Stoeckert CJ, Roos DS (2003). OrthoMCL: identification of ortholog groups for eukaryotic genomes. Genome Res.

[49] Wang XW, Luan JB, Li JM, Bao YY, Zhang CX, Liu SS (2010). *De novo* characterization of a whitefly transcriptome and analysis of its gene expression during development. BMC Genomics.

[50] Elmer KR, Fan S, Gunter HM, Jones JC, Boekhoff S, Kuraku S, Meyer A (2010). Rapid evolution and selection inferred from the transcriptomes of sympatric crater lake cichlid fishes. Mol Ecol.

[51] Osada N, Hashimoto K, Kameoka Y, Hirata M, Tanuma R, Uno Y, Inoue I, Hida M, Suzuki Y, Sugano S, Terao K, Kusuda J, Takahashi I (2008). Large-scale analysis of *Macaca fascicularis* transcripts and inference of genetic divergence between *M. fascicularis* and *M. mulatta*. BMC Genomics.

[52] Swanson WJ, Yang Z, Wolfner MF, Aquadro CF (2001). Positive Darwinian selection drives the evolution of several female reproductive proteins in mammals. Proc Natl Acad Sci U S A.

[53] Li JM, Su YL, Gao XL, He J, Liu SS, Wang XW (2011). Molecular characterization and oxidative stress response of an intracellular Cu/Zn superoxide dismutase (CuZnSOD) of the whitefly, *Bemisia tabaci*. Arch Insect Biochem Physiol.

[54] Yin Y, Martin J, Abubucker S, Scott AL, McCarter JP, Wilson RK, Jasmer DP, Mitreva M (2008). Intestinal transcriptomes of nematodes: comparison of the parasites *Ascaris suum* and *Haemonchus contortus* with the free-living *Caenorhabditis elegans*. PLoS Negl Trop Dis.

[55] Altschul SF, Madden TL, Schaffer AA, Zhang J, Zhang Z, Miller W, Lipman DJ (1997). Gapped BLAST and PSI-BLAST: a new generation of protein database search programs. Nucleic Acids Res.

[56] Yang Z, Nielsen R (2000). Estimating synonymous and nonsynonymous substitution rates under realistic evolutionary models. Mol Biol Evol.

[57] Zhang Z, Li J, Zhao XQ, Wang J, Wong GK, Yu J (2006). KaKs_Calculator: calculating Ka and Ks through model selection and model averaging. Genomics Proteomics Bioinformatics.

[58] Audic S, Claverie JM (1997). The significance of digital gene expression profiles. Genome Res.

[59] Benjamini Y, Yekutieli D (2001). The control of the false discovery rate in multiple testing under dependency. Ann Stat.

[60] Livak KJ, Schmittgen TD (2001). Analysis of relative gene expression data using real-time quantitative PCR and the 2(−Delta Delta C(T)) Method. Methods.

[61] Su Y, He WB, Wang J, Li JM, Liu SS, Wang XW (2013). Selection of endogenous reference genes for gene expression analysis in the Mediterranean species of the *Bemisia tabaci* (Hemiptera: Aleyrodidae) complex. J Econ Entomol.

