# Supplementary figures and images for: Transcriptomic analyses reveal the adaptive features and biological differences of guts from two invasive whitefly species
Source: BMC Genomics. 2014 May 15;15(1):370. doi: 10.1186/1471-2164-15-370 (PMC4035086; doi:10.1186/1471-2164-15-370)

Figure S1

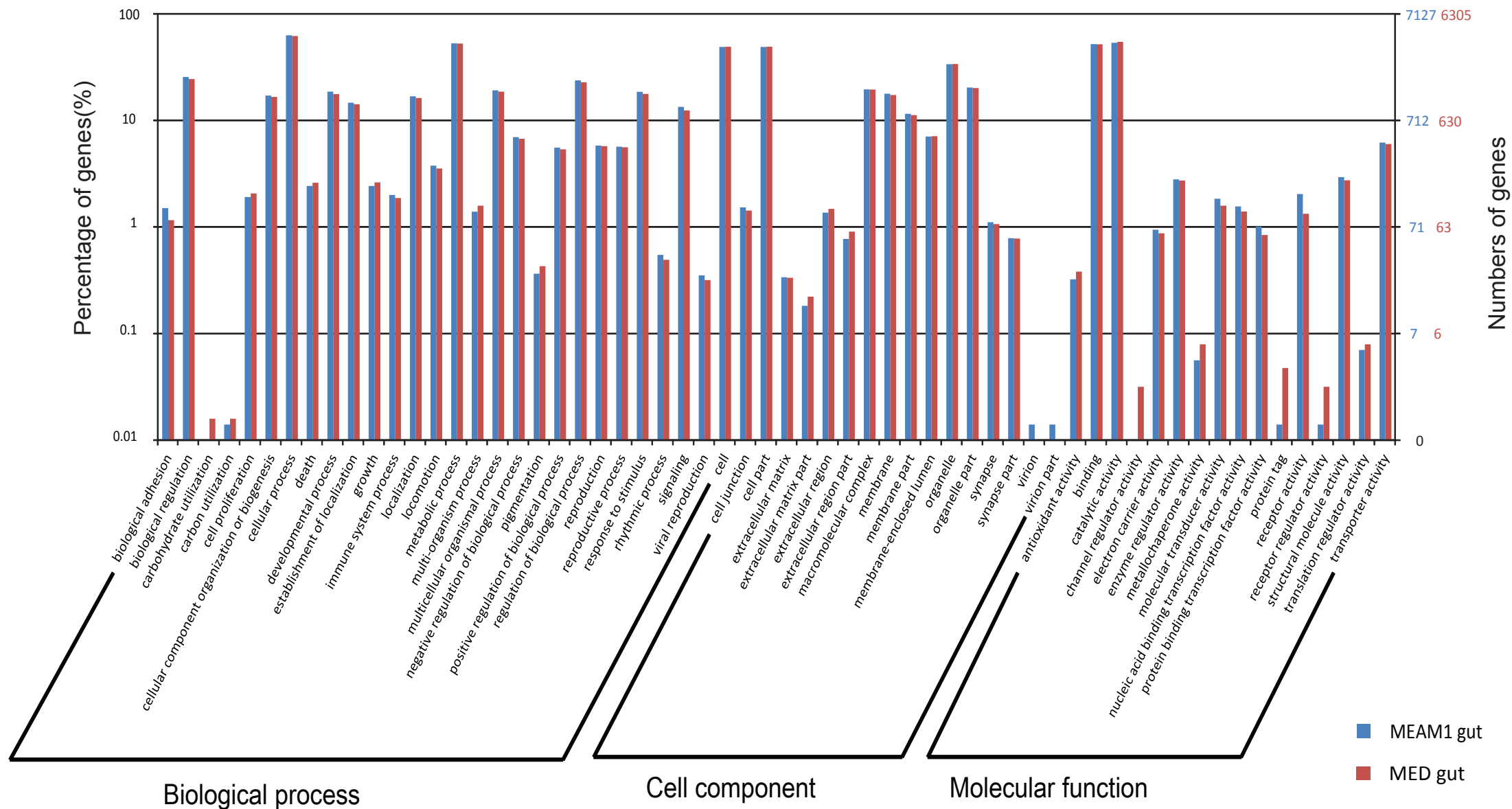

Supplement: Supplementary file 3 — Additional file 3: Histogram presentation of GO classification of genes from the MEAM1 and MED gut transcriptomes. The results are summarized in three main categories “Biological process”, “Cellular component” and “Molecular function”. The right y-axis indicates the number of genes in a category and the left y-axis indicates the percentage of a specific category of genes in that main category. (PDF 2 MB) [file 12864_2014_6066_MOESM3_ESM.pdf]

Figure S2

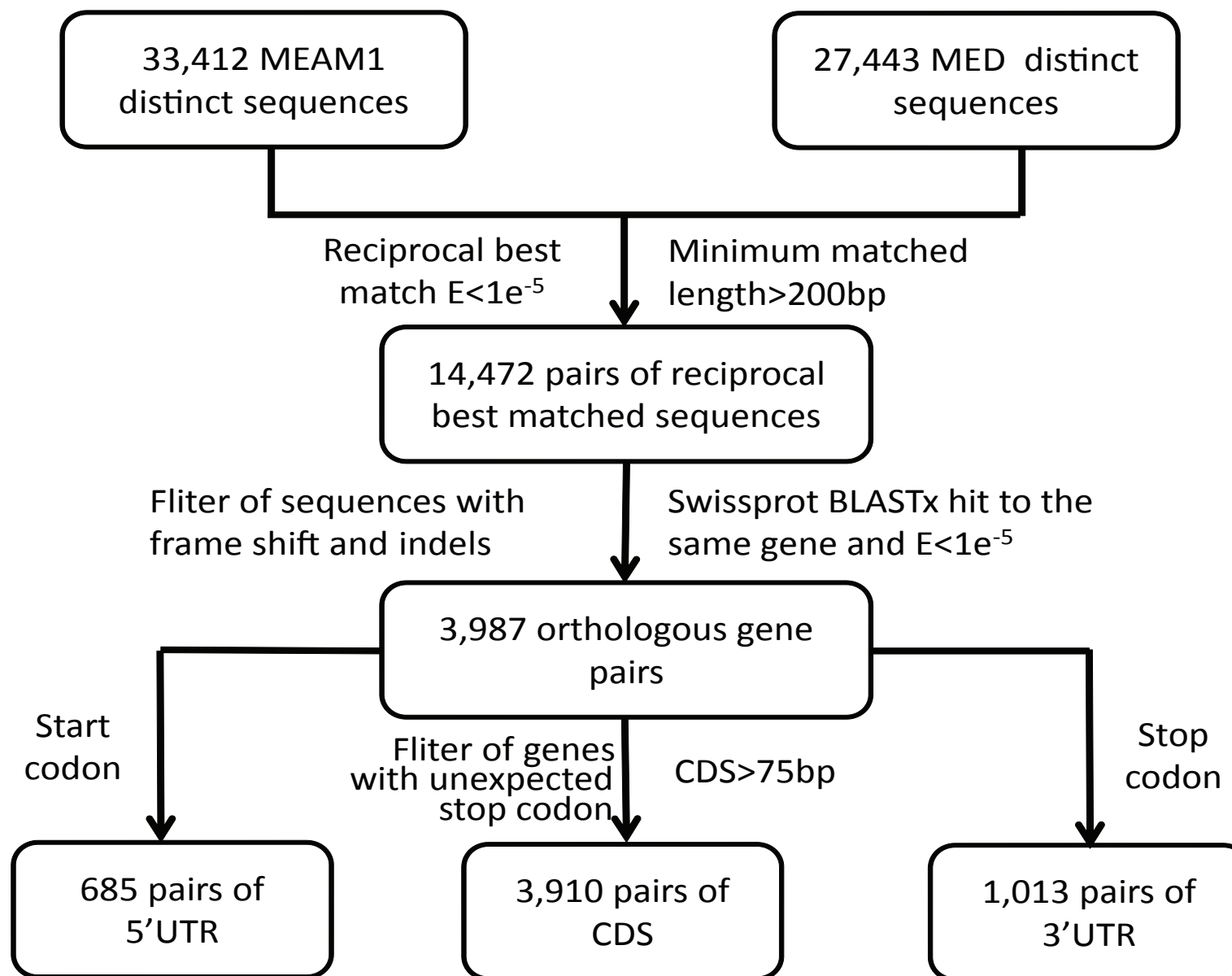

Supplement: Supplementary file 5 — Additional file 5: Identification and analysis of the orthologous genes between the gut transcriptomes of MEAM1 and MED. The orthologous genes were identified by bidirectional best hit method using MegaBLAST. All putative orthologs were further filtered against the Swissprot database. The putative orthologs that hit to different genes in the Swissprot database were removed. The CDS of the orthologous genes were determined by BLASTx against the Swissprot database with a threshold E-value of 1.0E−5. After removing the UTR regions, sequences shorter than 75 bp and with unexpected codons in the CDS were filtered. (PDF 314 KB) [file 12864_2014_6066_MOESM5_ESM.pdf]
